# The Life Science Exchange: a case study of a sectoral and sub-sectoral knowledge exchange programme

**DOI:** 10.1186/s12961-016-0105-4

**Published:** 2016-04-27

**Authors:** Brian Lee Perkins, Rob Garlick, Jodie Wren, Jon Smart, Julie Kennedy, Phil Stephens, Gwyn Tudor, Jonathan Bisson, David V. Ford

**Affiliations:** Health Informatics Group, Swansea University Medical School, Swansea, SA2 8PP Wales United Kingdom; Division of Psychological Medicine and Clinical Neurosciences, Cardiff University School of Medicine, Cardiff, CF14 4XN Wales United Kingdom; Wound Biology Group, Cardiff Institute of Tissue Engineering and Repair, School of Dentistry, College of Biomedical and Life Sciences, Cardiff University, Cardiff, CF14 4XY Wales United Kingdom; MediWales, The Bonded Warehouse, Cardiff, CF10 4HF Wales United Kingdom

**Keywords:** Knowledge exchange, Mapping sectoral, Innovation, Policy, Life sciences, Wales, Methods

## Abstract

**Background:**

Local and national governments have implemented sector-specific policies to support economic development through innovation, entrepreneurship and knowledge exchange. Supported by the Welsh Government through the European Regional Development Fund, The Life Science Exchange® project was created with the aim to increase interaction between stakeholders, to develop more effective knowledge exchange mechanisms, and to stimulate the formation and maintenance of long-term collaborative relationships within the Welsh life sciences ecosystem. The Life Science Exchange allowed participants to interact with other stakeholder communities (clinical, academic, business, governmental), exchange perspectives and discover new opportunities.

**Methods:**

Six sub-sector focus groups comprising over 200 senior stakeholders from academia, industry, the Welsh Government and National Health Service were established. Over 18 months, each focus group provided input to inform healthcare innovation policy and knowledge mapping exercises of their respective sub-sectors. Collaborative projects identified during the focus groups and stakeholder engagement were further developed through sandpit events and bespoke support.

**Results:**

Each sub-sector focus group produced a report outlining the significant strengths and opportunities in their respective areas of focus, made recommendations to overcome any ‘system failures’, and identified the stakeholder groups which needed to take action. A second outcome was a stakeholder-driven knowledge mapping exercise for each area of focus. Finally, the sandpit events and bespoke support resulted in participants generating more than £1.66 million in grant funding and inward investment. This article outlines four separate outcomes from the Life Science Exchange programme.

**Conclusions:**

The Life Science Exchange process has resulted in a multitude of collaborations, projects, inward investment opportunities and special interest group formations, in addition to securing over ten times its own costs in funding for Wales. The Life Science Exchange model is a simple and straightforward mechanism for a regional or national government to adapt and implement in order to improve innovation, skills, networks and knowledge exchange.

## Background

Following the creation of the National Assembly for Wales in 1998, the devolved Welsh Government (WG) has instituted a string of innovation initiatives in an attempt to invigorate the Welsh knowledge-based economy [1]. Nevertheless, despite receiving €3.9 billion from European Structural Funds since 2000, the Welsh economy has not improved its relative regional disparities in income, wealth and opportunities when compared to the United Kingdom as a whole and the European Union [2].

Many strategies exist for carrying out knowledge exchange within the life science (LS) sector, yet there is a lack of real-world application and evaluation of these interventions in the context of academic engagement and commercialisation [3]. Interventions aiming to improve innovation “*have met with varying levels of success, and, interestingly, the most prominent approaches have been, on the whole, less successful in Wales*” [4]. Successes in knowledge exchange activities include developing a shared perspective, a plan for collaboration, and a range of competencies [5].

In March 2012, the WG launched ‘Science for Wales’ [6], which set out a vision for where Wales should be heading towards in the future. Three ‘Grand Challenge’ priority areas where Wales has a track record for excellence were identified – ‘Life Sciences and Health’ being one of these. It has been demonstrated that the LS sector can drive economic growth through ‘multiplier effects’ while also generating outputs which benefit society and human health [7]. One of the key features of the LS sector is that the collaboration between actors within academia, industry, government and the National Health Service (NHS) is particularly vital in the transfer of research benefits to the public [8], which highlights the importance of having a coherent framework, strategy and policy which enables a thriving and sustainable LS sector.

In September 2012, Swansea University Medical School, in partnership with Cardiff University School of Medicine and MediWales (a membership organisation representing the LS industries of Wales), was tasked by the WG to lead a knowledge exchange project (KEP) for the Welsh Life Sciences and Health Grand Challenge priority area called The Life Science Exchange® (or LSX). The aim of the KEP was to increase interaction, develop more effective knowledge exchange mechanisms, and stimulate the formation and maintenance of long-term collaborative relationships between industry and academia.

The LSX was created as a process, not an organisation. The vision was to create a mechanism which could continuously network all the stakeholders in Wales’ complex LS ecosystem, in a way that allowed all to be involved. The objective was to allow participants to interact with other stakeholder communities (clinical, academic, business, governmental), exchanging perspectives, and then to support them by thinking about how Wales could improve its performance. To provide focus, the work was streamed into groups representing six of Wales’ specialist areas (Diagnostics, eHealth, Medical Technology, Neuroscience, Pharmaceuticals, and Regenerative Medicine), which were selected to encompass the 12 LS areas prioritised in ‘Science for Wales’ policy.

The LSX project was designed with a goal of having real world impact by supporting knowledge exchange, policy development and collaborative projects. The aims of this article are to articulate the methodologies used by the LSX project, provide case studies of collaborative projects identified and facilitated, and suggest how these methodologies could be applied to other sectoral or regional innovation systems.

## Methods

### Project overview

**Fig. 1:**
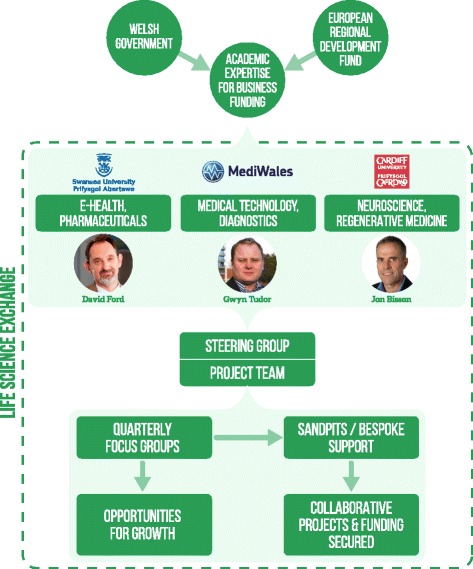
Overview of the Life Science Exchange Funding, Governance and Operations

In line with best practice for knowledge management projects [9], the LSX was overseen by a 17-member executive level steering group, comprising senior representatives of all stakeholder groups within the LS sector, including academia, business, NHS Wales, WG and third sector. The steering group convened quarterly over the 18-month project to review progress made at the focus groups and sandpit events. This group set the strategy for the project, agreeing the areas in which detailed research should be undertaken and the approach to be taken by the project team. The work of the focus groups and overall project management of the LSX was undertaken by a small project team led by Professor David Ford (see Fig. 1 for governance structure).

The LSX team comprised four staff members who provided overall project administration. Beyond planning, organising and documenting the focus groups and sandpit events, the team provided bespoke support to LSX members, including answering general inquiries regarding the Welsh LS sector, making introductions, identifying potential funding streams for collaborative projects, assisting with grant applications and mapping of the sector.

Given the aims, participant profiles and number of participants as well as the relatively informal manner in which discussions were held in these sessions, they were not ‘focus groups’ as understood in qualitative research. However, using the term ‘focus groups’ during and after the project allowed both participants and non-participants to quickly understand the activities that were being undertaken. After the end of the LSX project, some of these groups continued to meet and were more properly referred to as ‘special interest groups’.

### Focus group recruitment

For each of the six specialist areas, a focus group was established which aimed to include participants from academia, industry, the WG, industrial networks and NHS Wales. The project was funded with £181,553, of which a majority (£139,424) was used to support the staff organising the network communications and events over the 18-month project. Once the staff were recruited, they organised the branding, communications and organisation of the first focus groups to be held. The project team adopted an open and inclusive recruitment process for the groups. The LSX was widely publicised across the sector through established networks (e.g. the National Institute of Social and Health Research (NISCHR WG, now Health and Care Research Wales), MediWales, NHS Health Boards), as well as by using phone calls, e-mails, face-to-face meetings, social media (e.g. LinkedIn and Twitter), leaflets and other networking events. Additionally, the project team engaged in sector research early into the project to target individuals and organisations. The aim of this was to give all the major sector stakeholders a fair chance to feed into the process and to create groups that covered the interests of their specialist area as broadly as possible.

### Focus group meetings

Each partner institution chaired two focus groups on a quarterly basis. The purpose of the focus groups was to act as the primary information gathering mechanism for the project – primarily for developing policy recommendations, performing a knowledge mapping exercise and to promote collaborative working across the sector. Members of the group were invited to regular, facilitated discussions which captured stakeholder perspectives on the strengths, weaknesses and opportunities for growth across the LS sector. As well as taking this broad approach, specific focus groups also identified areas that were unique to their field of interest.

Focus group attendance ranged from 10 to 40 stakeholders, depending on the subsector, time of year, availability of the individuals, and geographic location of the meeting. The focus groups were audio-recorded and transcribed, which provided a meticulous and robust capturing method whilst allowing the project team to focus on the discussions [10]. Similar to other policymaking dialogue initiatives [11], Chatham House Rules [12] were adopted in order to create an environment where participants could speak freely. To ensure that the LSX was as inclusive as possible, the project team also engaged with some organisations and individuals on a one-to-one basis. This option was offered to those who wanted to participate in the process but did not necessarily have the time or were geographically too far away to engage through the focus group programme. In most cases, the project team met these stakeholders in person and discussed their perceptions of the LS sector. In other cases, these discussions were held over the phone or via email communication. The results of these individual consultations were included into the data which informed the final report writing and knowledge mapping.

As the groups developed, the members were encouraged to consider which interventions might be put in place to counteract sector weaknesses and capitalise on existing strengths. An outcome of each focus group session was a detailed record of the main conclusions that had been agreed upon. These were used to inform the final summary and subsector reports which were produced and circulated to WG’s Departments of Economy, Science and Transport and of Health and Social Services to consider for action.

For example, towards the end of the project, the Regenerative Medicine focus group had the objective to critically consider a strength, weakness, opportunity and threat (SWOT) analysis of the regenerative medicine landscape within Wales in order to produce a final recommendation for future development of this sector across the academic, clinical and commercial Welsh research landscape. A one-day focus group was organized in Cardiff, bringing together representatives from across the academic, clinical and commercial regenerative medicine settings. To position thinking in a wider United Kingdom context, initial presentations from Innovate UK (formerly the Technology Strategy Board) and Cell Therapy Catapult were received. This was followed by a comprehensive update of the findings from previous LSX Regenerative Medicine focus groups to that point in the form of a SWOT analysis. Break out groups then considered, in detail, shortfalls, problems and solutions to stimulate the regenerative medicine sector in Wales. Feedback was collected and outputs produced for feedback to the LW KEP management board.

### Knowledge mapping

The project team and stakeholders instigated a knowledge mapping exercise of their respective specialty areas similar to the process described by Ebener et al. [13]. The project team engaged with higher education institutions, NHS contacts, industry contacts and the WG to identify existing network maps and information sources across the sector. These were then cross referenced and validated to ensure they were current additions to the knowledge mapping and not either obsolete or a previous incarnation of another current body. This served to inform businesses, academics and other stakeholders of the institutional, community and policy level networks.

Knowledge mapping was performed to provide a comprehensive visual map and database of expertise and facilities across academia, NHS and industry relevant to each sub-sector within Wales. The mapping was undertaken using a five-step process:

1. Scoping: The knowledge mapping exercise began by identifying potential data sources. The project partners provided the LSX project team access to a range of existing networks and also knowledge bases of the sector. These included academic centres and NHS research and development centres, as well as Welsh government and industrial contacts. The project team then cross referenced these sources.
2. Data collection: Information was gathered from the data sources identified during scoping.
3. Validation: Verification checks were made by team members to ensure the information supplied was accurate.
4. Collation: The mapping was collated by both the LSX project team and MediWales and presented in both visual and database formats suitable for searching by keyword.
5. Gap analysis and review: The mapping was reviewed by all the focus groups and also peer examined by other academic, NHS, industrial networks and WG Expertise Wales. Any gaps identified were addressed to ensure the final mapping was reflective of the sector.

The databases contained the names, affiliated institution, contact information, web-link and a short description of the individual/group expertise which could be probed by keyword. Academic researcher and medical team data was gathered from institute websites and email contact with departmental leaders. Industry data was compiled from MediWales’ Picture of Health [14], online research and provided by focus group members. General LS mapping provided data on WG and NISCHR initiatives and teams within the sector, societies operating within Wales and identified awards of excellence held by Welsh researchers.

### Sandpit events

The LSX engaged in a series of half-day sandpit events which came about in response to opportunities raised through the focus groups. Sandpits were originally designed by the United Kingdom Research Councils as a residential interactive workshop over 5 days involving 20–30 participants with a multidisciplinary mix of participants to drive lateral thinking and radical approaches to address research challenges [15]. The project team assessed the range of opportunities that had been discussed at different stages of the project. Opportunities which were most likely to interest a broad range of stakeholders were considered as the focus for these half-day sandpit events, which brought together a wider audience from within the LS sector to feedback on the findings of the focus groups and to input additional information and ideas. The main objective of these events was to explore the possibilities for collaboration within the event-specific opportunity area, as well as provide the opportunity to have a more in-depth discussion about key topics identified through the focus groups.

One example of the sandpit approach was focused on the objective of ‘developing approaches to address unmet needs in the area of managing chronic illness in ageing’ within the Medical Technology group. This event was a collaborative opportunity for leading academics, medical practitioners, service managers and industry representatives in Wales to consider which chronic diseases were not met within the NHS, where expertise may exist in Wales and whether participants in the sandpit were collaboratively equipped to enable a technology to manage a met condition better or address an unmet disease. The purpose of the first part of the sandpit was to familiarise the group with the participants on the day, their interests and background, and to define a list of 10–12 illnesses that cannot be cured but can be managed. The sandpit participants, in multidisciplinary groups, then examined this list to consider the expertise, experience and interests of the group to explore the opportunities to create collaborative proposal(s) for future work. The final part of the sandpit was looking at scoping initial collaborative ideas to address the objective of the event and presenting them. The 40 participants were drawn from across academia, industry and the NHS in Wales and worked together in small multi-disciplinary teams to maximise shared experience and knowledge with the aim of generating creative solutions to known issues through partnership working. By the end of the day the participants explored their interests in collaborating with a number of different groups/individuals with the outcome of championing a single idea in a multi-disciplinary group for onward funding proposal preparation and work. This sandpit led to the preparation of funding proposals as an output.

### Bespoke support results

The LSX project was able to support the Welsh LS sector by a range of mechanisms. These activities resulted in a variety of outcomes for the sector, including detailed policy recommendation reports delivered to the WG, a codified mapping exercise of the LS sector in Wales, and a range of collaborative projects.

### Policy recommendations

A global overview of the opportunities identified in the focus groups have been categorised into seven major themes – each opportunity represented by a mark in Table 1.

**Table 1:** Summary of opportunities provided by each focus group. Each opportunity identified in each focus group is represented by a single dot. For example, the Neuroscience focus group provided four separate recommendations relating to enhanced research capacity

Opportunities	Diagnostics	eHealth	Medical technology	Neuroscience	Pharmaceuticals	Regenerative medicine
Establishment of new special interest groups			•	•	•	•
Mapping, sector knowledge and communication		•	•		•	•
NHS procurement and technology adoption	•	•	•		•	
NHS engagement and clinical access	•	••	•		•	•
Growing a skilled and trained workforce	•		•	•	•	
Enhanced research capacity	•	•		••••		•
International conferences and marketing	•				•	•

There was a recognised need for the discussions instigated by the LSX to continue into the future. A number of organisations expressed the desire to maintain the momentum of their respective focus groups as special interest groups.

The need for an accessible, cost-effective, up-to-date source of sector-specific information relating to funding, regulation, international trade and market intelligence has been consistently expressed by the stakeholder focus groups. This represents a significant and ongoing opportunity to provide Welsh LS organisations with a valuable resource offered in addition to what is (and is not) available.

The vital role that the Welsh NHS plays in providing access to clinical expertise, facilities and, ultimately, as a customer was widely expressed. While the relationship between the NHS, academia and industry in Wales is considered to be a significant national strength, there was an expressed need to improve the evaluation and adoption of new Welsh innovations when they can demonstrate the opportunity for improved patient outcomes and cost savings. Furthermore, academics and industry expressed a strong desire for NHS Wales to increase its level of engagement and receive clinical access for research and development.

A key driver for future success is the ability to attract and retain highly skilled individuals. Whilst the higher education sector can focus on the development of training and, where feasible, accredited education in key skills, retention requires growing industrial small and medium enterprise clusters to provide sustainable employment opportunities across Wales. An all-Wales LS skills group consisting of key academics has organised discussions on delivering apprenticeships, continuous professional development and degree-level training in close collaboration with WG, NHS Wales and LS industry.

Stakeholders felt that significant strengths in Wales’ LS research activity have been demonstrated across the entire Welsh LS sector. They recommended that, in order to build upon these strengths, further support through provision of facilities and resources was required to close specific gaps in research capacity.

The focus groups also made a strong case for ongoing support for international trade and promotion. Most LS sector products occupy global markets and numerous Welsh companies sell the majority of their products outside of Wales. To support this activity, focus groups requested continued or additional support to attend trade shows and conferences.

### Knowledge mapping

A second outcome was a stakeholder-driven knowledge mapping exercise for each area of focus. Many examples of duplication of activity as well as underutilisation of resources were identified. In some instances, businesses were paying to utilise resources outside of Wales due to a lack of awareness of resources existing locally.

The outputs of the knowledge mapping exercises for each focus group were represented in various formats, including a database of key figures, lists of key organisations, process diagrams, organisational charts and maps. For example, the eHealth focus group produced a database outlining key individuals in WG, learned societies, industry, academic departments, and research facilities. This database included institution and/or department names, named individuals, websites and contact details which were publicly available. The eHealth group also created a set of PowerPoint slides containing organisational charts of academic, WG, NHS Wales and third sector organisations. Similarly, the Regenerative Medicine focus group produced a database containing academic, commercial and NHS Wales networks and facilities. As the group was research-focused, they also created an organisational chart of regenerative medicine research by technology type (stem cells, disease process and biomarkers, cell signalling, etc*.*).

### Funding secured

Although funded with only £181,553, the LSX project was able to secure over £1.66 million pounds of project funding for its participants through the sandpit events and bespoke support:

- £800,000: Innovate UK, Small Business Research Initiative (SBRI), Abertawe Bro Morgannwg University Health Board (ABMUHB)
- £772,208: WG, Health Technology and Telehealth Fund AliveCore, ABMUHB
- £50,000: Welsh Government Academic Expertise For Business Digital Health Conference
- £40,000: Betsi Cadwallder SBRI SymlConnect

### Case studies

Beyond quantitative financial outputs, a range of case studies have been provided below in order to describe the types of outcomes the LSX was able to accomplish beyond the focus groups and knowledge mapping. Such outcomes included the piloting of new procurement mechanisms, securing grant funding for a commercial clinical trial, establishment of new special interest groups, and the formation of an all-Wales LS Skills Group, among others.

#### New procurement mechanisms

One of the projects the eHealth group identified was the 3-year SBRI, funded by Innovate UK, which aims to boost the research and development industry, increase commercialisation of new technologies and create jobs and wealth. A project to improve ABMUHB, in partnership with Swansea University, has received £800,000 funding towards their £950,000 project to support health services in Swansea, Bridgend and Neath Port Talbot through the better use of patient records and data.

The SBRI project came about following discussions in an eHealth focus group, which was led by a member who had prior experience of undertaking SBRIs outside of Wales. Also at that meeting was the manager from the eHealth Industries Centre in Swansea University, who subsequently identified the SBRI opportunity and engaged with ABMUHB. ABMUHB identified a challenge and were extremely receptive and supportive of the SBRI process. The eHealth group then discussed the opportunity and many attended the two events hosted by LSX with eHealth Industries Innovation Centre (ehi^2^) and WG.

Two local companies were tasked with using patient data to shape the future of patient care in the region. They were the strongest candidates to emerge from the feasibility phase of a 3-year SBRI launched in partnership with ABMUHB and Swansea University’s Health Informatics group. Together, these companies have worked to help ABMUHB better understand the health needs of patients, and predict how these could change. This could support much more efficient use of resources, and ultimately help improve public health services; as well as the health and well-being of local communities.

#### New commercial clinical trial

One funding source identified in the eHealth focus group was the WG’s £9.5 million Health Technology and Telehealth Fund. A United States-based company, AliveCor, manufacture heart monitors that can be used with most mobile technology. In January 2014, the WG introduced AliveCor to the LSX project team, who provided bespoke support to form a collaborative project between the company and eHealth focus group attendees. Staff at Swansea University’s Joint Clinical Research Facility are now working with Morriston Hospital, the WG and AliveCor to trial the AliveCor system, which converts iPhone and Android compatible mobile devices into hand-held ECG machines that can be used to monitor a person’s heart. This research is funded by WG’s Health Technology and Telehealth Fund. It is proposed that the AliveCor system will be trialled in the clinic to identify patients with atrial fibrillation in the community to facilitate earlier diagnosis and treatment and reduce the incidence of major adverse cardiovascular events, notably stroke.

The LSX team continued to contribute to the project by assisting with the governance, project management, value creation and economic value sections of the bid, as well as obtaining formal confirmation of support from the ABMUHB Research and Development department. In April 2014, the £652,208 bid to the Health Technology and Telehealth Fund was approved, with AliveCor making an additional £120,000 inward investment into Wales.

#### New commercial special interest groups

MediWales was introduced to a range of pharmaceutical stakeholders at a LSX pharmaceutical focus group event in February 2014. Directly following these interactions, MediWales facilitated a LSX pharmaceutical sandpit event in June 2014. The event, hosted at the Norgine factory in Hengoed, was attended by 23 delegates from 13 companies, universities and healthcare providers, all of whom are involved in pharmaceutical research, drug development, formulation, manufacture, packaging, trials or distribution. As a result of the meeting the group identified an opportunity to work together to deliver and promote a full range of services to the pharmaceutical sector. Under the brand name Clinical Trials Services Wales, with ongoing support from MediWales, the new special interest group will continue to meet regularly. They are now developing a brand identity, mapping clinical trials services in Wales against the pharmaceutical route to market and developing marketing, promotional material and a programme of activity for the coming year.

The concept was driven by the idea that MediWales members involved in the delivery of clinical trials can meet international customer needs from ‘molecule to market’ and together represent a significant global force. Through collaboration, the interest group member organisations have identified the opportunity to (1) provide a faster more streamlined service to their customers, (2) to create new business opportunities, and (3) to deliver a complete clinical trials service and to support their customers’ needs through a strong referral network. Following this launch, the Members will work together to deliver on a commitment to provide a more integrated streamlined service, saving customers time and money, and delivering the highest quality of service. The Group will work together to extend each organizations visibility on the world stage through promotion at international events, press coverage and online. Each individual organisation will act as a referral point to all of the members of the Group.

This ‘sub-sector’ approach is now being rolled out to create a medical technology and diagnostics special interest group led by MediWales.

#### New all-Wales life sciences skills group

Within a diagnostics focus group, skills for the sector were identified as an area where development was required. The LSX project team subsequently met with representatives from Cogent, the Sector Skills Council for Life Science, as well as WG’s Department for Education and Skills and Department of Economy, Science and Transport. There was a level of involvement from the Sector Skills Council for Science, Engineering and Manufacturing Technologies, University of South Wales and the Regional Learning Partnership.

An all-Wales LS skills group organised discussions on delivering apprenticeships, continuing professional development and degree-level training in close collaboration with WG, NHS Wales and the LS industrial sector. The group, now chaired by a representative from Swansea University Medical School, has been recognised by the WG for its proposed work on the LS skills agenda and aims to represent the needs of the growing sector in this arena.

## Discussion

The LSX project delivered extensive engagement with the Welsh LS sector in order to provide authentic insight into the strengths, challenges and opportunities for Wales. The significant contribution made by representatives from the Welsh LS academic, industrial and clinical organisations has culminated in the final reports of the LSX project.

### Policy recommendations

Detailed conclusions have been made by each of the sector focus groups, with a number of common and unique themes across all of them. Based on the varying outputs of recommendations by each focus group, it is clear that each of the specialties has different needs. The stakeholders in the Neuroscience Group appeared to be more active in earlier stage research and development and recognised opportunities in enhancing Wales’ neuroscience research capacity. Those that have more of an industrial base (e.g. eHealth, Diagnostics, Medical Devices and Pharmaceuticals) focused their attention on NHS engagement, procurement and technology adoption. All of the summarised opportunities found in Table 1 were discussed in each focus group; however, some were not identified as a recommendation to address for their respective specialty.

The detailed reports have been supplied to the WG’s LS Sector Team to inform policy and strategy; however, it is unclear just how effective these recommendations will be until future policy is implemented. They were also made available to key stakeholders by the WG upon request. These recommendations provide the best opportunity for the sub-sector groups to progress their work as they provide the unified view of the current state of the Welsh LS sector and how it should be improved in their areas of speciality. In current discussions with stakeholders, these recommendations are being used to inform research and funding applications, especially in the establishment of new special interest groups, growing a skilled and trained workforce, and enhanced research capacity (i.e. equipment and facilities).

### Knowledge mapping

The value of the exercise is difficult to quantify, though recipients of the mapping have utilised it to inform new academic- and industry-based knowledge sources of their own. It could be stated that the exercise provided a clearer sector picture of a diverse range of mapping and knowledge already in existence and therefore gave organisations who received it an up to date and accurate ‘snapshot’ of the LS sector in Wales for others to build on. In a fast moving sector this was as much as could be expected as the accuracy and completeness of any mapping exercise lessens as time passes.

The knowledge mapping exercises were supplied to the WG’s LS Sector Team to inform policy and strategy. The maps were developed to cover four constituent areas, those being academia, industry, NHS and Government (including allied services such as MediWales). These maps were then focused further on the theme (e.g. medical technology, regenerative medicine or eHealth) to provide a snapshot of subsectors of LS in Wales. They were made available to NISCHR, LSX participants, LS Hub Wales and key stakeholders by the WG upon request. MediWales has since utilised the mapping to inform their ‘Picture of Health’ [14] interactive map of the Welsh LS sector. NISCHR have utilised the mapping to inform their 2015 service structure review and it has also been utilised by the LS Hub. Academic use included Swansea and Cardiff Universities.

### Limitations

However successful, a major drawback of the LSX was the short 18-month time frame and limited budget to carry out its operations. The timeline and budget was solely based on the project obtaining funds from the end of the European Regional Development Fund structural funds programme running from 2007 to 2014. Following the end of the project, further actions were required to ensure its legacy was exploited. The subsector focus groups were able to codify their detailed reports and mapping exercises by the end of the project. However, without the project staff to organise the network after the report submissions, there was very little that could be done by the LSX support staff to ensure the policy recommendations were addressed by the WG. Furthermore, it was difficult to resource collaborative projects identified towards the end of the project, as there was a risk of leaving them without support. If LSE had been funded for a longer period of time or refunded after the initial period, the project could have had more impact on the LS sectoral policy in Wales. As a recommendation, sustainability and/or continuity planning should be performed at the beginning of projects such as the LSX in order to ensure that value in the personnel, networks and project(s) are not lost between funding rounds.

### Adaptation of LSX

The LSX was focused on supporting the LS sector in Wales in a subsector approach. Notwithstanding this, the framework could be applied to a very wide range of other (sub)sectors and/or regions. Simply setting up a knowledge exchange mechanism similar to the LSX would not necessarily result in successful or similar outcomes – each sector and region has its own particularities that would need to be considered. For example, although two other KEPs were funded by the WG concurrently to the LSX, they did not follow the same governance and methods. Instead of holding focus groups, the Materials and Advanced Engineering Knowledge Exchange Strategy KEP determine the sector’s barriers and opportunities compared to 30 companies outside Wales. Through these methods, this KEP was able to secure two Knowledge Transfer Partnerships valued at £129,000, placed about £500,000 in collaborative grant applications and created a range of Masters-level studentships with industry. The Low Carbon Energy and Environment Network for Wales KEP used a mixture of workshops, surveys and research to report on the major barriers and opportunities for the low carbon energy and environment sector in Wales; yet it is unclear how successful their model was in producing collaborative projects.

The implementation of a successful knowledge exchange programme requires a number of factors to be in place. First and foremost, a relatively active sectoral innovation system is needed to ensure enough stakeholders can contribute to the process. Secondly, project leaders need to have relatively established networks in place to ensure effective and timely recruitment. Thirdly, dedicated staff and an event budget are needed to ensure the network is able to communicate, meet regularly and synthesise their knowledge. Finally, in order for meaningful and innovative collaborative projects to be supported beyond the conception stage, robust funding mechanisms are required (e.g. Innovate UK or the United Kingdom Research Councils).

## Conclusions

The output of the LSX is a valuable body of intelligence that represents the collective expertise of a wide range of expert contributors. This work should serve to inform future policy and planning across the sector and will help to align support activities with the needs of companies, universities and healthcare providers. Specific, actionable recommendations have been provided in the detailed reports provided by the LSX to the WG. Ultimately, this work should be used to improve innovation, health and wealth in Wales, as well as influence the design and implementation of knowledge exchange mechanisms in other geographies and sectors.

There was a recognised need for the discussions instigated by the LSX to continue into the future. In some cases, specific challenges and opportunities need to be crystallised into detailed proposals with specific objectives, deliverables, budgets and time-scales. A number of organisations have expressed the desire to maintain the momentum of their respective focus groups as special interest groups operating under the LSX brand or unique branding (e.g. Clinical Trials Services Wales).

The LSX process succeeded in bringing together hundreds of stakeholders in a sub-sectoral approach to the Welsh National Innovation System. This has resulted in a multitude of collaborations, projects, inward investment opportunities, and special interest group formations, in addition to leveraging over ten times its funding for Wales. Processes such as the LSX can be considered exemplars of best practice for knowledge exchange for other sectoral systems of innovation. The LSX model is a simple and straightforward mechanism for any regional government to adapt and implement with the hopes of improving innovation, skills, networks and knowledge exchange.

## Abbreviations

ABMUHB: Abertawe Bro Morgannwg University Health Board
KEP: Knowledge exchange programme
LS: Life science(s)
LSX: Life Science Exchange
NHS: National Health Service
NISCHR: National Institute of Social and Health Research
SBRI: Small business research initiative
WG: Welsh Government
